# Bilateral Synchronous Sporadic Renal Cell Carcinoma: Retroperitoneoscopic Strategies and Intermediate Outcomes of 60 Patients

**DOI:** 10.1371/journal.pone.0154578

**Published:** 2016-05-02

**Authors:** Baojun Wang, Huijie Gong, Xu Zhang, Hongzhao Li, Xin Ma, Erlin Song, Jiangping Gao, Jun Dong

**Affiliations:** Department of Urology, Chinese PLA General Hospital/Chinese PLA Medical Academy, Beijing, P. R. China; UNIFESP Federal University of São Paulo, BRAZIL

## Abstract

**Objective:**

To evaluate the presentation, management, pathology, and functional and oncological outcomes of patients undergoing retroperitoneoscopic treatment of bilateral synchronous sporadic RCC at our institution.

**Methods:**

We retrospectively evaluated the records of 60 patients with bilateral synchronous sporadic RCC who underwent retroperitoneoscopic treatment at the General Hospital of People's Liberation Army from 2008 to 2014. The estimated glomerular filtration rate was calculated and compared among different surgical procedures. The overall survival and recurrence free survival were assessed based on information from recent follow-up.

**Results:**

Fifty-six patients underwent bilateral retroperitoneoscopic surgeries in staged procedures, and four patients underwent bilateral retroperitoneoscopic surgeries in simultaneous procedures. Among the former group of patients, 34 underwent bilateral partial nephrectomy, 12 underwent radical nephrectomy followed by partial nephrectomy, and 10 underwent partial nephrectomy followed by radical nephrectomy. Bilateral partial nephrectomy can better preserve renal function (p = 0.040) and the sequence of partial nephrectomy and radical nephrectomy did not affect functional outcomes (p = 0.790). One patient undergoing simultaneous procedures developed acute renal failure and required temporary hemodialysis. At 3 and 5 years, overall survival rates were 93.0% and 89.4%, and recurrence free survival rates were 90.5% and 81.6%. High nuclear grade (p = 0.014) was related to disease recurrence.

**Conclusions:**

Staged bilateral partial nephrectomy was efficient in preserving renal function. The survival of patients with bilateral synchronous sporadic renal tumors was similar to that of patients with unilateral nonmetastatic tumors. Nuclear grade was an independent prognostic factor of disease recurrence.

## Introduction

Bilateral renal tumors remain relatively uncommon, accounting for 1%- to 5% of patients with renal cell carcinoma (RCC) [1–3]. Those with bilateral synchronous sporadic RCC are a distinct subpopulation and have a different biological behavior from hereditary bilateral RCC.

Surgery is the method of choice to treat bilateral sporadic RCC, because it has a comparable prognosis to that of unilateral sporadic RCC [4,5]. The awareness that chronic kidney disease and/or rapid decline in estimated glomerular filtration rate (eGFR) increases the risk of cardiovascular events and death is growing [6]. Minimizing treatment-related loss of renal function is of particular importance for bilateral renal tumors. Balancing the need for complete eradication of potentially malignant tissue with the goal of maximal functional preservation of the bilateral synchronous RCC presents a challenge.

Given that the information about clinical features and retroperitoneoscopic management of bilateral synchronous sporadic renal cell carcinoma (BSSRCC) is limited, we evaluated the clinic pathological features and prognosis of patients undergoing retroperitoneoscopic resections of BSSRCC from 2008 to 2014 in our institution. Surgical options include retroperitoneoscopic partial nephrectomy (RPN) and retroperitoneoscopic radical nephrectomy (RRN). Moreover, we compared the perioperative eGFR changes in patients undergoing different surgical procedures.

## Materials and Methods

This retrospective study was conducted with a surgery database at PLAGH that is approved by the institutional review board. Written Informed Consent was obtained from all patients. This study was approved by the Protection of Human Subjects Committee, PLAGH. Between 2008 and 2014, of the patients who presented with bilateral renal tumors, 60 met the criteria for BSSRCC and were surgically treated at our institution, which were selected for analysis. Those with known hereditary syndromes and those who underwent surgery on only one side, were excluded. Given that patients with multifocal lesions undergoing RPN with relatively longer warm ischemia time (WIT), which could affect functional results, and the unclarified mechanism of this kind of RCC, we excluded these patients. All patients underwent simultaneous surgery or staged surgery on both kidneys within a 1-year period.

Preoperative clinical evaluation consisted of physical examination, chest X-ray, and abdominopelvic CT or MRI. All of the 60 patients were treated with bilateral retroperitoneoscopic surgeries. The surgical approach of RRN or RPN to BSSRCC is determined by the comorbidity of the patient, tumor status, and surgeon expertise. For the retroperitoneoscopic procedure, we preferred a 3-trocar technique for the operation as previously described [7–9].

Clinical data of the patients including age, gender, BMI, and the presence of the disease were collected and the detailed information was provided in S1 Table. The complexity of tumors was measured by the preoperative aspects and dimensions used for an anatomical (PADUA) classification of renal tumours[10]. Pathological data included T classification, nuclear grade, and histological subtype [11]. Tumors were staged or graded in either kidney with the higher tumor stage or grade.

Renal functional evaluation was conducted by using the sCr values obtained a day before the initial and second surgery, a day after initial and second surgery, and 3–6 months after the second surgery. The Chronic Kidney Disease Epidemiology Collaboration (CKD-EPI) creatinine equation was used to calculate eGFR, accounting for patient age, race, gender, and sCr level [12]. The difference and percentage change of perioperative eGFR were calculated to evaluate the effect of the different surgical procedures. Postoperative CKD staging was conducted according to the National Kidney Foundation Disease Outcomes Quality Initiative Clinical Practice Guidelines [13]. CKD stage was defined as stage I (≥90 ml·min^-1^·1.73 m^-2^), stage II (60–89 ml·min^-1^·1.73 m^-2^), stage III (30–59 ml·min^-1^·1.73 m^-2^), stage IV (15–29 ml·min^-1^·1.73 m^-2^), and stage V (<15 ml·min^-1^·1.73 m^-2^).

For postoperative oncological follow-up, an abdominal CT or MRI, chest X-ray and bone scan as a baseline within 3–6 months after surgery were recommended, then annually during the first 5 years and every 2 years thereafter[14]. End points for oncological follow-up included disease recurrence and death, and the 5-year OS and RFS were calculated. Recurrence is defined as radiographically verified local recurrence or progression to nodal or distant metastasis during the study period.

Statistical analysis was performed by using Empower Stats version 2.13. The association between clinicopathological features and tumor behavior was examined by using univariable and multivariable cox proportional hazards regression models. Kaplan–Meier analysis was conducted to calculate percent survival at specific times. Differences in survival were compared by using the log rank test. All tests were two sided with statistical significance determined at p < 0.05.

## Results

Demographic data and Tumor characteristics are shown in Table 1. Of the 60 patients with BSSRCC, 48 (80.0%) were men and 12 (20.0%) were women with a median age of 50 years. The clear cell RCC was the main subtype, accounting for 89.2% (107/120) of the kidney tumors involved. Bilateral clear cell RCC was determined in 80.0% (48/60) of patients.

**Table 1:** Patients, tumors and subsequent clinical behavior.

	value
**No. of patients**	60
**Median age at first surgery (range)**	50(25–69)year
**Median BMI (range)**	26(15–35)kg/m^2^
**Gender**	
No. of men (%)	48(80.0%)
No. of women (%)	12(20.0%)
**Median tumor size (range)**	4.4(1.4–10)cm
**Median PADUA score (range)**	8(6–14)
**No. of renal cell carcinoma**	120
No. of clear cell (%)	107(89.2%)
No. of papillary (%)	7(5.8%)
No. of chromophobe (%)	3(2.5%)
No. of sarcoid (%)	3(2.5%)
**Tumor stage**	
No. of pT1a (%)	35(58.3%)
No. of pT1b (%)	16(26.7%)
No. of pT2a (%)	9(15.0%)
Nuclear grade*	
No. of G1 (%)	51(85.0%)
No. of G2 (%)	7(11.7%)
No. of G3 (%)	2(3.3%)
**Post-op CKD stage**	
No. of stage I	5(8.3%)
No. of stage II	34(56.7%)
No. of stage III	21(35.0%)
**Outcomes**	
No. of no evidence of disease (%)	49(81.7%)
No. of local recurrence (%)	1(1.7%)
No. of metastasis (%)	4(6.6%)
No. of dead (%)	6(10.0%)
**Median interval days(range)**	59(0–238)day
**Median follow-up (range)**	43(7–82)month

CKD = chronic kidney disease

*Nuclear grade G1: Fuhrman grade I + II; Nuclear grade G2: Fuhrman grade III; Nuclear grade G3: Fuhrman grade IV.

All 60 patients underwent successful retroperitoneoscopic resection of bilateral tumors without any conversion to open surgery. For patients undergoing RPN, no positive margin was determined. 56 patients undergoing bilateral surgeries in a staged manner were grouped based on sequential RPN (RPN-RPN, 34), RRN followed by RPN (RRN-RPN, 12), and RPN followed by RRN (RPN-RRN, 10). Four patients underwent bilateral surgery in a simultaneous manner, bilateral RPN in one patient, RRN followed by RPN in two patients, and RPN followed by RRN in one patient.

The median interval time for two surgeries was 59 (0–238) days. No significant difference existed in age, gender, body mass index, and preoperative eGFR among groups. Tumor size, PADUA score and WIT were similar for patients treated with RPN in all groups. In the RPN-RPN group, the first surgery was conducted for the larger tumor in 68% of cases, and median tumor diameter was 3.5 cm in the first RPN versus 2.7 cm in the second RPN (p = 0.09). The median PADUA score value was also 1 point higher (8 to 7) in the first RPN than in the second RPN. Median tumor diameters in RRN and RPN were 5.5 cm and 3.0 cm, respectively (p < 0.01).

The PADUA scores of the 120 kidney tumors were summarized and categorized according to surgical procedures and sequences in Table 2. The median values of the PADUA score were 11 and 7 in RRN and RPN, respectively. And in the first and second surgery, the median PADUA score values were 9 and 7, respectively.

**Table 2:** Preoperative aspects and dimensions used for an anatomical (PADUA) classification of renal tumors treated with different surgical procedures and sequences.

	Surgical procedure		Surgical sequence	
	RRN	RPN	First	Second
**No.(%)**	25(20.8%)	95(79.2%)	60(50%)	60(50%)
**PADUA score**				
6–7	2(1.6%)	49(40.9%)	17(14.2%)	34(28.3%)
8–9	3(2.5%)	45(37.5%)	30(25.0%)	18(15.0%)
10–14	20(16.7%)	1(0.8%)	13(10.8%)	8(6.7%)

**Fig 1:**
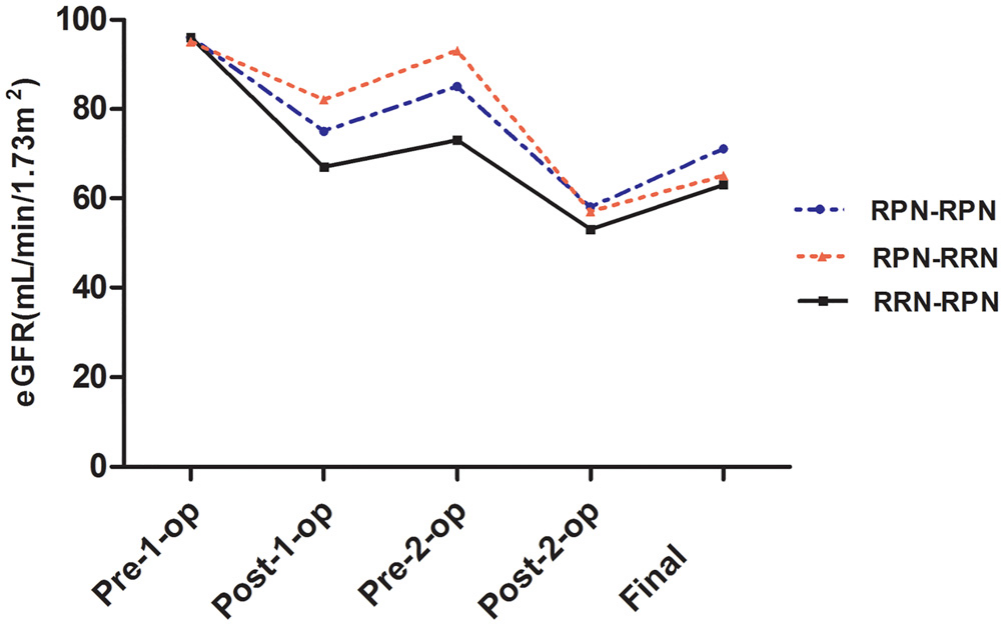
Renal functional changes for different surgical procedures. The patients of RPN-RPN, RRN-RPN and RPN-RRN groups exhibited 22%, 30% and 17% decrease rates in eGFR undergoing the first operation, and 32%, 29%, and 45% undergoing the second operation, respectively.

Of the four patients undergoing simultaneous bilateral surgeries, one patient undergoing RRN-RPN developed acute renal failure with sCr 707.1 μmol/L. Renal insufficiency occurred in the other two patients with the sCr level 189.3 and 357.1 μmol/L after RPN-RPN and RPN-RRN, respectively. After immediate hemodialysis, the patient with acute renal failure recovered, and the long-term functional outcome was comparable with that of staged groups with the eGFR stable at around 50 ml·min^-1^·1.73 m^-2^. The median WIT of simultaneous surgery was 21 (11–26) min compared with the median WIT 23 (10–40) min of all the RPN conducted. The tumor characteristics were also comparable to those of staged groups. For the staged group, the eGFR data are summarized in Table 3. The trends of perioperative eGFR changes are shown in Fig 1. The patients of RPN-RPN, RRN-RPN and RPN-RRN groups exhibited 22%, 30% and 17% decrease rates in eGFR undergoing the first operation, and 32%, 29%, and 45% undergoing the second operation, respectively. The final eGFR results show that bilateral RPN can better preserve renal function (p = 0.040), and the order of RRN and RPN did not influence the final functional result (p = 0.790).

**Table 3:** Renal functional changes of stage procedure*.

	RPN-RPN	RRN-RPN	RPN-RRN	P Value**
**No.**	34	12	10	
**Mean eGFR(SD)(ml·min**^**-1**^**·1.73 m**^**-2**^**)**				
Pre-1-op	96(11)	96(11)	95(13)	0.991
Post-1-op	75(15)	67(16)	79(16)	0.157
Pre-2-op	85(14)	73(15)	89(16)	0.025
Post-2-op	58(17)	52(13)	49(11)	0.248
Final	71(16)	63(10)	59(8)	0.040

eGFR = estimated glomerular fitration rate, RRN = retroperitoneoscopic radical nephrectomy, RPN = retroperitoneoscopic partial nephrectomy, SD = standard deviation

*Patient undergoing bilateral RRN was excluded for analysis.

**Evaluates differences among surgery groups using Kruskal Wallis test.

For the oncological outcomes, 49 patients survived without tumor, 5 patients developed local recurrence or metastasis, and 6 patients died. The median (range) follow-up of the whole cohort was 43 (7–82) months. At 3 and 5 years, the OS rates were 93.0% and 89.4%, and RFS rates were 90.5% and 81.6%, respectively. Table 4 shows the univariate and multivariate Cox regression analysis for disease recurrence in the patients surgically treated with BSSRCC. High nuclear grade (HR 6.3, p = 0.006), high T stage (HR 8.4, p = 0.002) and larger tumor size (HR 1.5, p = 0.004) were significant predictors on univariate analysis. After adjusting for age at surgery, BMI, gender, PADUA score, postoperative CKD stage, and surgery type, only high nuclear grade was the independent prognostic factor for disease progress (HR 32.4, p = 0.014). Surgery type (HR 4.8, p = 0.319)/ (HR 15.6, p = 0.587) and PADUA score (HR 1.33, p = 0.279) did not affect survival. For the OS, all the parameters failed to reach statistical significance, although 3 of the 5 patients died with high T stage or high nuclear grade.

**Table 4:** Univariate and multivariate cox regression analysis predicting disease recurrence in patients treated with bilateral retroperitoneal laparoscopic surgeries.

	univariate analysis		multivariate analysis	
	HR (95%CI)	P Value	HR (95%CI)	P Value
**Gender**				
Male	1.0		1.0	
Female	1.3(0.28, 6.30)	0.729	1.2(0.25, 18.69)	0.495
**Age**	1.0(0.94, 1.10)	0.958	1.0(0.92, 1.15)	0.595
**BMI**	1.0(0.81, 1.20)	0.876	1.0(0.71, 1.29)	0.756
**Surgery type**				
RPN/RPN	1.0		1.0	
RRN/RPN	2.2(0.45, 10.30)	0.338	4.8(0.11, 41.94)	0.319
RPN/RRN	2.7(0.65, 11.50)	0.168	15.6(0.63, 195.48)	0.587
**Nuclear grade**				
G1	1.0		1.0	
G2+G3	6.3(1.70, 23.90)	0.006	32.4(2.02, 520.25)	0.014
**TNM stage**				
T1N0M0	1.0		1.0	
T2N0M0	8.4(2.30, 31.60)	0.002	1.5(0.17, 13.70)	0.703
**Tumor size**	1.5(1.10, 2.00)	0.004	2.2(0.90, 5.25)	0.086
**PADUA score**	1.3(0.99, 1.10)	0.065	1.33(0.45, 1.26)	0.279
**Interval days**	1.0(0.98, 1.00)	0.180	1.0(0.99, 1.02)	0.901
**Post-op CKD stage**				
Stage I	1.0		1.0	
Stage II	0.6(0.05, 7.00)	0.685	0.7(0.04, 12.01)	0.831
Stage III	7.6(0.66, 86.50)	0.104	6.7(0.2, 206.99)	0.276

HR = hazard ratio, CI = confidence interval, BMI = body mass index

## Discussion

This report represents our experience of managing BSSRCC with retroperitoneoscopic surgery. Sixty patients met our criteria for BSSRCC and were treated with retroperitoneoscopic surgery on both sides. The key finding of this study was that staged bilateral RPN was superior in renal functional preservation with equivalent oncological results. Simultaneous bilateral surgeries had a high risk of postoperative renal dysfunction.

Nephron-sparing surgery is the treatment standard for patient with bilateral renal tumors. Laparoscopic PN has improved significantly and offered advantages of less operative time, decreased operative blood loss, less ischemia time, and fewer complications with equivalent renal functional and oncological outcomes, despite the increasing tumor complexity, such as large, completely endophytic or hilar masses [15,16]. All these advantages made the surgical treatment of bilateral renal tumors feasible. In our series, all 60 patients underwent successful retroperitoneoscopic resection of bilateral tumors without requiring a traditional open procedure, of which 35 (58%) patients underwent bilateral RPN. Increased use of bilateral PN occurred in the management of bilateral renal tumors [17,18]. Simmons et al [18] analyzed the cumulative data from 220 patients with bilateral renal tumors, of which 134 (61%) patients underwent sequential PN. Lowrance et al [17] reported 44% of his cohort underwent bilateral PN, which has increased over time for improved functional outcome and comparable oncological outcome.

No absolute consensus exists on staged or simultaneous procedure and on which kidney to be operated first. At our center, the preference is for staged bilateral PN 35 (58%) and for operating on the more complicated mass (with higher PADUA score) first. We have several reasons to operate in this way. First, patients who underwent simultaneous surgeries would suffer bilateral operative trauma, thereby running an increased risk of acute renal insufficiency, and some even required temporary hemodialysis for treatment. However, in a staged procedure, if the large tumor was resected first, the contralateral kidney can function as a backup instead of being traumatized during the operation, which minimizes the chance of dialysis [19,20]. Second, a staged procedure allows the patient to alter the treatment strategy for the second renal lesion based on the pathological findings and outcomes of the first surgery. Given that disease progression has increased chance to happen on tumor with a high stage, the resection of the large tumor would provide us more related information. Third, the unpredictable perioperative complications, such as postoperative bleeding or urine leak, would be easy for doctors to manage and safe for patients during a staged procedure [20].

Most clinical centers take the staged approach as a routine procedure. The Memorial Sloan Kettering Cancer Center series preferred staged partial nephrectomy and operating on the more involved kidney first [1,17,21]. And some other clinical centers explained the preferred surgical strategy based on individual scenarios [22,23]. The mayo clinic carried out a series of reports that about 70% of the patients were treated in a simultaneous transperitoneal procedure and that they preferred operating on the kidneys with more complex tumors first. Nevertheless, they did not deny the feasibility and efficiency of the staged procedure [2,4,5].

This study also evaluated functional changes in patients undergoing bilateral surgery. Our result indicated that patients who underwent a simultaneous procedure would take an increased risk of renal dysfunction. Bilateral-staged RPN is the method of choice to preserve renal function with the final mean eGFR (SD) at 71 (16) ml·min^-1^·1.73 m^-2^. Functional outcomes have no difference regardless of whether RRN or RPN is the initial procedure in the RRN-RPN group. The outcomes of patients treated with staged surgical procedures are difficult to compare with the outcomes of those with simultaneous procedures because only four patients underwent simultaneous procedures. Blute et al [2,4] reported in their series that the patients who underwent bilateral surgery in a single procedure can obtain excellent prognosis with acceptable early functional results, but they did not detail the functional changes in their reports. Simmons et al [18] analyzed a group of 220 patients undergoing sequential bilateral kidney surgery and showed that bilateral PN is associated with significantly improved eGFR compared with RN-PN and that patients with good postoperative renal functions obtain improved OS.

The data from current cohort showed midterm oncological results: OS rates were 93.0% at 3 years and 89.4% at 5 years, and RFS rates were 90.5% at 3 years and 81.6% at 5 years, comparable with results of documented reports [3,5]. High nuclear grade was associated with low RFS but not low OS, which indicated that noncancerous factors contributed to patient survival and that long follow-up time was needed. Other academic centers presented their results with a relatively long follow-up time for patient survival. Simmons et al [18] reported OS rates of 86% at 5 years and 71% at 10 years and RFS rates of 73% at 5 years and 44% at 10 years. Boorjian et al [5] reported an OS of 51% and a CSS of 70% at 10 years in 92 patients. Klatte et al [3] showed that the OS rates at 5 years and 10 years are 87% and 78%, and that these rates have no difference compared with those of unilateral nonmetastatic RCC.

Traditionally surgical exploration and partial or total nephrectomy without preoperative histological diagnosis is the standard procedure for supposed malignant renal mass management. This routine procedure is accepted due to the high frequency of malignant disease when solid renal masses are discovered during radiological explorations. Performing percutaneous biopsies for renal tumors remains a controversial issue. Several studies of renal biopsy have demonstrated high sufficiency and accuracy. Sufficiency for diagnosis has been reported at 79%-100% and the accuracy has been 86%-95.5% with relatively low complication rates [24,25]. A significant proportion of patients experience a change in clinical management after biopsy, which is salient for small renal masses, defined as those <4 cm in diameter. Especially bilateral renal masses to choose the most conservative therapy are strong indications for preoperative biopsy [26]. However, according the guideline of the Chinese Urological Association, renal mass biopsy was generally not indicated for healthy patients, and was limited to exclude metastasis to kidney, lymphoma, or infection, and was infrequently used as a routine management. Surgical removal of renal mass is still common practice, even though some benign lesions were thought to be malignant and being surgically resected. At our present series, we focused on the surgical strategy, and none of those patients underwent preoperative biopsy. Given the precision of the diagnosis and the relatively low morbidity associated with the procedure, renal mass biopsy should be considered as a part of the algorithm for choosing the best therapy in the management of small renal masses.

A centrally held, de-identified nephrectomy registry allows countries to benchmark nephrectomy performance and refine the use of the procedure through research [27]. However, China currently has no such registry for renal tumors. We believe that the nephrectomy registry that collects data for both partial and radical procedures would aid in the monitoring of follow-up practice as well as the development and refinement of the RCC management and a national nephrectomy registry will be built in the near future.

The limitations of this study include the retrospective study design, the time of renal function measurement among groups, and the limited sample number in specific subgroups. The eGFR was calculated with the CKD-EPI creatinine equation based on the sCr levels, which might not reflect the functional changes accurately. The effect of postoperative CKD on OS was attenuated by the limited follow-up time. However, we compared the eGFR changes among different surgical procedures. Although we used the PADUA score to evaluate the anatomical complexity of renal tumors, we didn’t associate it with the functional outcomes of the patients treated with bilateral RPN. For bilateral renal masses, preoperative biopsy should be thoroughly considered. Further study with a larger population, of which patients treated with RPN should be subdivided according to PADUA score, will be meaningful.

## Conclusions

The staged retroperitoneoscopic procedure is safe, effective, and feasible in managing bilateral RCC. Staged RPN is the strategy of choice whenever possible, because it can obtain equivalent oncological outcomes with optimal preservation of renal function. OS and RFS in patients with BSSRCC are equivalent to those of patients with unilateral nonmetastatic renal tumors. High nuclear grade is independent prognostic factor of BSSRCC, and bilateral RPN does not increase the risk of disease recurrence and death.

## Ethical Standards

Written Informed Consent was obtained from all patients. This study was approved by the Protection of Human Subjects Committee, Chinese People’s Liberation Army (PLA) General Hospital.

## Supporting Information

S1 Table Data set of 60 patients. (XLS)

## Abbreviations

BMI: body mass index
BSSRCC: bilateral synchronous sporadic renal cell carcinoma
CKD-EPI: the Chronic Kidney Disease Epidemiology Collaboration
eGFR: estimated glomerular filtration rate
OS: overall survival
PADUA: Preoperative aspects and dimensions used for an anatomical
RFS: recurrence free survival
RCC: renal cell carcinoma
RPN: retroperitoneoscopic partial nephrectomy
RRN: retroperitoneoscopic radical nephrectomy
sCr: serum creatinine
WIT: warm ischemia time
